# Mr. Rafter and Lake Erie Water Supply

**Published:** 1896-11

**Authors:** William G. Bissell

**Affiliations:** Department of Health


### Correspondence.

### MR. RAFTER AND LAKE ERIE WATER SUPPLY.

*To the Editor:*

SIR—The August number of the BUFFALO MEDICAL JOURNAL contained an article On Lake Erie as a water supply for the towns on its borders, by George W. Rafter. On the twenty-first page were the following statements:

The Health Department of the City of Buffalo has, to the credit of that city, instituted frequent systematic bacteriological examinations of the Lake Erie water as supplied to the consumers in Buffalo. By courtesy of Dr. William G. Bissell, city bacteriologist, I have been afforded an opportunity to examine the results of the last year's work. The most interesting point brought out is the great decrease in number of bacteria present in the water as it leaves the reservoir in comparison to the number in the water as it enters. The sedimentation frequently reduces the number from 30,000 to 40,000 per cubic centimeter to from 400 to 700 per cubic centimeter. In a practical way, however, it ought not to be overlooked that the water at the bottom of the reservoir must contain vast numbers, among which it is probable that some are the specific morbific causes of disease. We may conclude, then, that the bottom needs to be cleaned frequently as a safeguard against possible contamination from this source.

The officials of the Buffalo Health Department are, however, disposed to say that Lake Erie is an unexceptionable source of water supply because they hardly ever detect the specific germ of typhoid fever. On this point I am compelled to disagree and cite the results of an investigation of the relation of the bacillus of typhoid fever to sewage made by Laws and Andrewes for the London County Council, as published in the early part of 1895.

Mr. Rafter then rehearses the generally conceded evidence in regard to the unreliability of typhoidal investigation with sewerage and the like.

Where the author (Mr. R.) obtained the positive information regarding the influence of sedimentation upon the water in the Buffalo reservoir I am at a loss to understand, for careful examination of the official records in the bureau of bacteriology does not reveal such information.

As to Lake Erie being an "unexceptionable source of water supply," (depending on the failure to demonstrate the presence of the typhoidal organisms,) the following letter, in answer to a personal inquiry as to where this information was obtained, fully explains the reliability of the statements as coming from the health department:

GEO. W. RAFTER, M. AM. SOC. C. E.,
EXPERT ENGINEER.

ROCHESTER, N. Y., August 30, 1896.

*My Dear Dr. Bissell :*

Yours of August 11th reached me in due course. I did not refer to you at all in the matter of special stress on inability to detect the typhoid bacillus. The statements published in the Buffalo papers, as coming from the department, are my source of information on the point in question.

You are safe in the proposition that Buffalo water is, at times, very seriously contaminated.

I hope to have the pleasure of talking with you again some time.

Very sincerely, GEO. W. RAFTER.

To DR. WM. G. BISSELL, Buffalo, N. Y.

It is a known and acknowledged fact that the public water supply of Buffalo is open to serious contamination at any time, and undoubtedly is more or less polluted all the time.

It is and has been the custom of the health commissioner and his subordinates to recommend constant household filtration.

WILLIAM G. BISSELL.

Department of Health, October 9, 1896.

